# Neoantigen‐reactive T cell: An emerging role in adoptive cellular immunotherapy

**DOI:** 10.1002/mco2.41

**Published:** 2021-04-23

**Authors:** Yicheng Zhu, Youkun Qian, Zhile Li, Yangyang Li, Bin Li

**Affiliations:** ^1^ Department of Immunology and Microbiology, Shanghai Institute of Immunology Shanghai Jiao Tong University School of Medicine Shanghai China

**Keywords:** immunotherapy, neoantigen, T cell, tumor

## Abstract

Adoptive cellular immunotherapy harnessing the intrinsic immune system for precise treatment has exhibited preliminary success against malignant tumors. As one of the emerging roles in adoptive cellular immunotherapy, neoantigen‐reactive T cell (NRT) focuses on the antigens expressed only by tumor cells. It exclusively obliterates tumor and spares normal tissues, achieving more satisfying effects. However, the development of NRT immunotherapy remains in a relatively primitive stage. Current challenges include identification of NRTs and maintenance of adoptive cell efficacy in vivo. The possible side effects and other limitations of this treatment also hinder its application. Here, we present an overview of NRT immunotherapy and discuss the progress and challenges as well as the prospects in this promising field.

## Graphical abstract

Neoantigen‐reactive T‐cell therapy is a thriving branch of immunotherapy in fighting against cancer. It only attacks tumor cells and spares normal tissue. There are different means to select T cells and various efforts could be made to enhance their in vivo application.

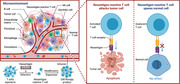

## 1 INTRODUCTION

Malignant tumors have elevated to the second leading cause of disability‐adjusted life years.^1^ In recent years, immunotherapy has stepped onto the center stage, which harnesses the immune system to fight against tumors. The spontaneous antitumor immune responses indicate the opportunities for intervention, including removing immunosuppression to restore antitumor effects such as immune checkpoint blockade (ICB),^2^ and using cancer vaccines to stimulate the body to attack tumors.^3^ Adoptive T‐cell therapy (ACT) is also an important part of immunotherapy. It works by extracting and screening specific T lymphocytes, then infusing them back after modification and amplification to mediate the tumor‐killing activity.^4^ Some currently used ACTs, such as chimeric antigen receptor T‐cell therapy (CAR‐T), exert the therapeutic actions by targeting antigens on the surface of tumor cells. These approaches have demonstrated excellent performance in hematologic tumors, but not so well in solid tumors, possibly incriminating the nonspecial targets or limited number of surface antigens.^5^

Therefore, neoantigens that are only expressed in tumor cells are research hotspots. With the latest advances in deep sequencing, machine learning prediction algorithms, and synthetic biology, many neoantigen‐reactive T cell (NRT)‐based immunotherapy modalities are launched. These recruits prevent the attacks on normal tissues caused by the nonspecificity (off‐target effect) and broaden the target spectrum as well. Attracted by its huge prospects, clinical trials focusing on a wide range of tumors are carried out in full swing (Table 1). It has been shown in recent clinical trials that metastatic breast cancer and colorectal cancer patients obtained objective remission after the administration of NRTs.^6^, ^7^

**TABLE 1:** Selected neoantigen‐reactive T cell clinical trials

Neoantigen	Identifier	Phase	Enrollment	Intervention	Disease condition	Recruitment status	Sponsor
NY‐ESO‐1	NCT01343043	I	50	Chemotherapy, NY‐ESO‐1(c259)T cells	Synovial sarcoma	Completed	GlaxoSmithKline
MAGE‐A3	NCT02111850	I/II	21	Chemotherapy, anti‐MAGE‐A3‐DP4 TCR PBL, aldesleukin	Cervical cancer, renal cancer, urothelial cancer, melanoma, breast cancer	Active, not recruiting	NCI
MAGE‐A4	NCT03132922	I	52	Autologous genetically modified MAGE‐A4(c1032)T cells, low‐dose radiation	Urinary bladder cancer, melanoma, head and neck cancer, ovarian cancer, NSCLC, esophageal cancer, gastric cancer, synovial sarcoma, myxoid/round cell liposarcoma	Recruiting	Adaptimmune
MART‐1	NCT00509288	II	24	Chemotherapy, aldesleukin, autologous anti‐MART‐1 F5 TCR gene engineered TIL	Skin cancer, metastatic melanoma	Completed	NCI
HERV‐E	NCT03354390	I	24	Chemotherapy, HERV‐E TCR transduced CD8+/CD34+ T cells	Kidney cancer	Recruiting	NHLBI
KRAS‐G12V	NCT04146298	I/II	30	Chemotherapy, mutant KRAS G12V‐specific TCR transduced autologous T cells, anti‐PD‐1 McAb	Pancreatic cancer	Recruiting	Guo ShiWei
KRAS‐G12D	NCT03745326	I/II	70	Chemotherapy, anti‐KRAS G12D mTCR PBL, aldesleukin	Gastrointestinal cancer, pancreatic cancer, gastric cancer, colon cancer, rectal cancer	Recruiting	NCI
Individualized	NCT03412877	II	270	Chemotherapy, individual patient TCR‐transduced PBL, aldesleukin, anti‐PD‐1 McAb	Glioblastoma, NSCLC, ovarian cancer, breast cancer, gastrointestinal cancer, genitourinary cancer	Recruiting	NCI

*Note*. Data updated on July 31, 2020 from clinicaltrials.gov.

Abbreviations: PBL, peripheral blood lymphocytes; TCR, T cell receptor; NCI, National Cancer Institute; NSCLC, nonsmall cell lung cancer; TIL, tumor infiltrating lymphocytes; NHLBI, National Heart, Lung, and Blood Institute; McAb, monoclonal antibody.

**FIGURE 1:**
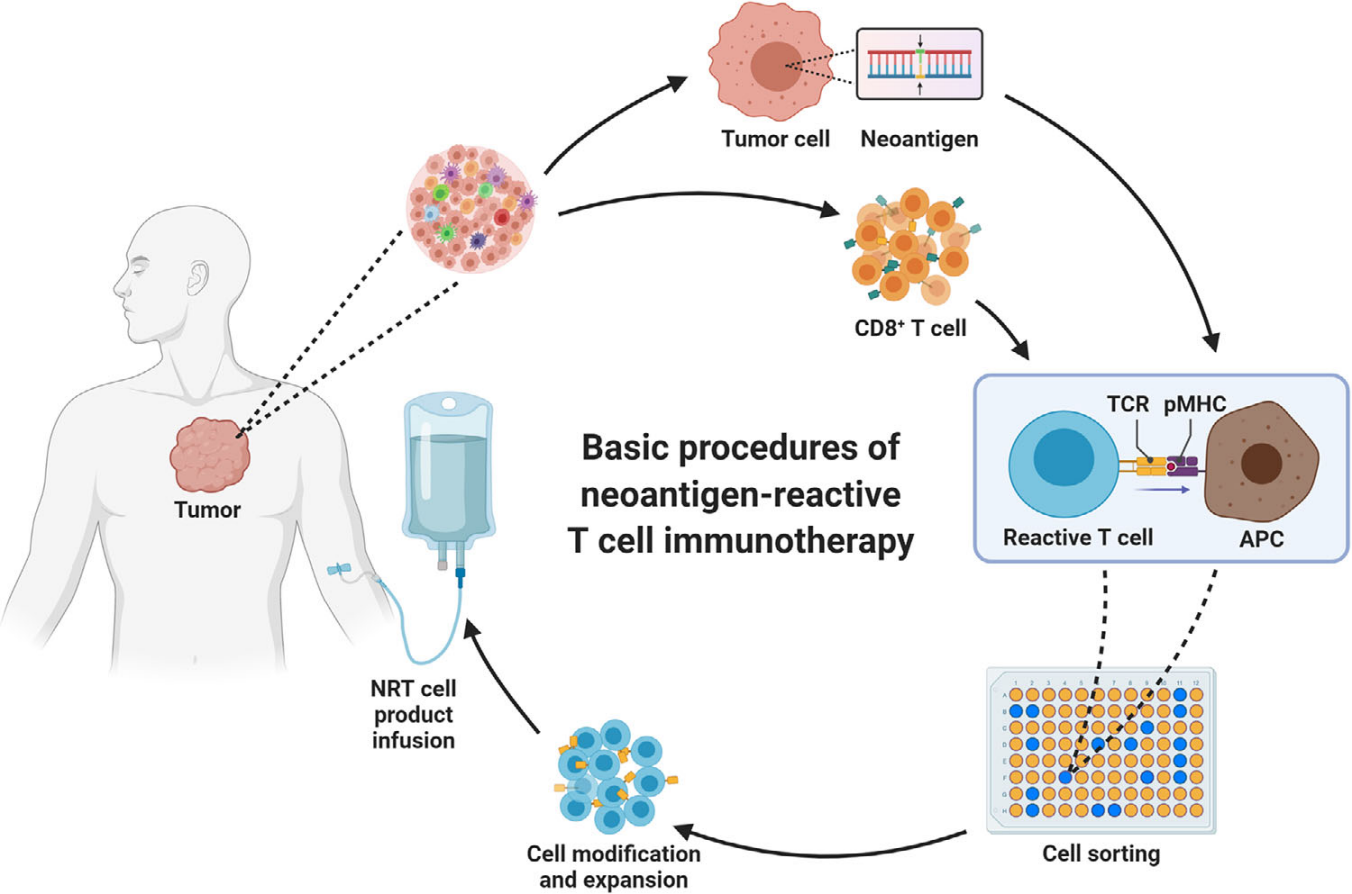
Basic procedures of neoantigen‐reactive T cell immunotherapy. Tumor cells and CD8+ T cells are first isolated from the patient. And the neoantigens in the tumor cells are identified by sequencing. Then neoantigen‐presenting cells are co‐cultured with the CD8+ T cells and neoantigen‐reactive T cells are thus sorted. After in vitro modification and expansion, the neoantigen‐reactive T cell product can be infused back to the patient

Despite the variety of forms, NRT immunotherapy can be disassembled into a few basic procedures: pinpointing the neoantigen and the reactive T cells, modifying them in vitro as needed, and reinfusing them back to the patient (Figure 1). However, due to the limited progress of the current study, our understanding of NRT therapy is still in the darkroom. Although some positive results bring us light, we should be soberly aware that there are many obstacles to be overcome. In this review, we introduce and analyze the general aspects of NRT therapy, discuss the potential challenges of each part, and summarize the latest progress in related fields.

## 2 IDENTIFY NRTs

NRTs have the potential to push the boundaries of cell therapy enormously because of their ability to distinguish tumor cells from normal cells by neoantigen recognition. Presently, the most challenging part of NRT therapy development is to identify and expand NRTs.

### 2.1 Putative neoantigen prediction

The first step in NRT therapy is to identify and select the putative neoantigen. Nonsynonymous variants were identified through comparing DNA or RNA sequences of tumor and normal cells. Putative neoantigens were further predicted by inputting these sequences of variants into the machine learning models, including NetMHC, NetMHCpan, and MHCflurry,^8^, ^9^, ^10^ which use algorithms to predict the peptide‐human leukocyte antigen (HLA) binding affinity. However, the antigen‐presenting procedure, which includes protein synthesis, proteasome degradation, HLA combination, and transportation, is complicated and still poorly understood. Now the in silico antigen prediction algorithms cannot cover all these and output a relatively high false‐positive result.^11^ Though the specificity of neoantigen prediction can be increased with the modified algorithms or more precise data collected from mass spectrometry,^12^, ^13^ it still cannot meet our expectations. Therefore, verification of putative neoantigens should be performed before experimental or clinical use.

### 2.2 Neoantigen verification

#### 2.2.1 Tandem minigenes and long peptides

**FIGURE 2:**
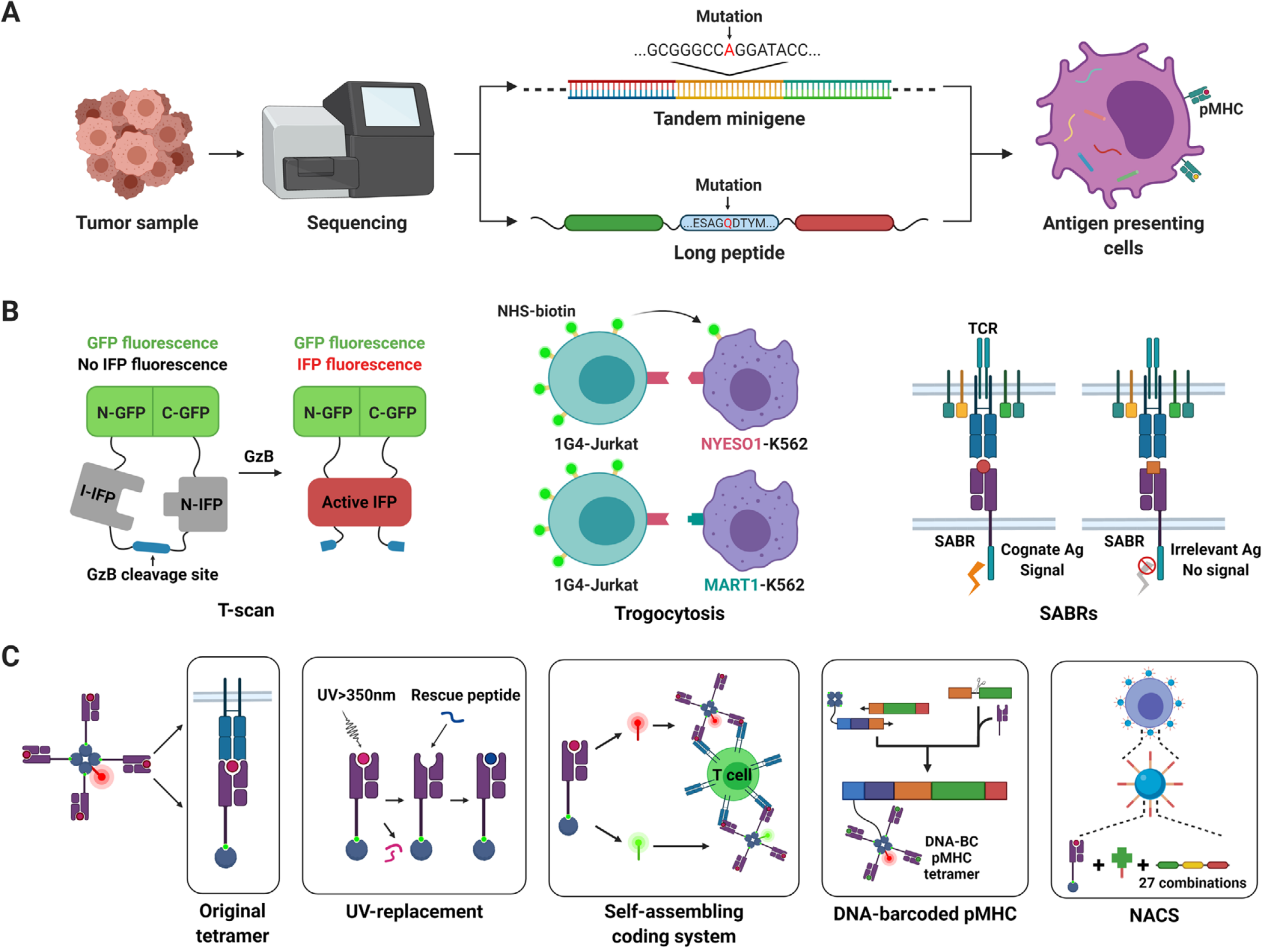
Basic approaches and the latest progress in identifying neoantigens. (A) After in silico prediction, TMGs and long peptides are synthesized, then transfected or pulsed into APCs to present neoantigens. (B) T‐scan platform loads the APCs with special reporters that can fluoresce after cleavage by enzymes released from T cells. Another method marks membrane protein on T cells with NHS‐biotin. While trogocytosis happens, the marked proteins are transferred to the recognized APCs. SABRs contain special domains and induce TCR‐like signal while interacted. (C) UV‐replacement strategy uses a conditional MHC ligand that can be cleaved by UV light and replaced by the peptide of interest. In the self‐assembling coding system, tetramers containing the same neoantigen are colored in different combinations (eg, combine red and green). DNA‐barcoded pMHC tetramer directly connects neoantigen DNA sequence with tetramer. Nanoparticle‐barcoded NACS system uses three docking sites that can bind labeled ssDNA in sequence (green, yellow, and red) to represent a neoantigen Abbreviations: APC, antigen presenting cell; MHC, major histocompatibility complex; NACS, nucleic acid cell sorting; NHS, *N*‐hydroxysuccinimide; SABR, signaling and antigen‐presenting bifunctional receptor; TMG, tandem minigene; UV, ultraviolet.

Neoantigens are mainly validated by monitoring the responses of tumor‐infiltrating lymphocytes (TILs) in co‐culture with the presented antigens. In the early days, antigen presentation was done by introducing neoantigen‐encoding genes into antigen presenting cells (APCs) that express specific HLA subtypes. This method applies the natural antigen presentation procedure, which substantially decreases the false‐positive hits compared with the in silico peptide prediction algorithms.^14^ But it could be clumsy and time‐consuming when there are lots of neoantigens on the list. The solution is to use tandem minigene (TMG) or long peptide instead (Figure 2A). The TMGs carry multiple minigenes, each containing a mutant nucleotide in the middle with normal nucleotides on each side, which encode a neoepitope. The long peptides are composed of multiple peptides, each of which has a mutant amino acid flank by normal amino acids. These two methods can provide many neoantigens in one cell, preserving the advantage in the previous one and improving the screening efficiency, which have been successfully practiced in clinical trials on gastrointestinal cancers, metastatic melanoma, and epithelial cancers.^15^, ^16^, ^17^

Yet, the method above is still far from perfect. TMGs may not equally express all the minigenes, because they may be influenced by their position in the three‐dimensional structure of the protein translated from TMG,^18^, ^19^ whereas long peptides elicit a weaker CD8+ T‐cell response compared with TMGs.^20^ Moreover, APCs may also form new antigens constructed by parts of the nearby minigenes. Nowadays, the in silico assistance can reduce the formation of such artificial antigens.^21^

Besides, most of the existing detection methods focus on finding activated T cell, but cannot identify the recognized neoantigens, and the use of TMGs or long peptides makes it even harder. To overcome this problem, new technologies have been developed (Figure 2B). The T‐scan platform loads the APCs with special reporters carrying a granzyme B (GzB) cleavage site blocking the integration of infrared fluorescent proteins. While attacked by T cells, the corresponding APCs are able to release fluorescent signals in the presence of GzB from T cells.^22^ Fluorescence‐activated cell sorting (FACS) is then used to productively select the recognized APCs. There are also systems that express a signaling and antigen‐presenting bifunctional receptors. The receptors contain an extracellular domain that presents peptide and major histocompatibility complex (MHC), with an intracellular domain that induces a T‐cell receptor (TCR)‐like signal.^23^ After recognized by T cells, the APCs will induce an intracellular signal and highly express fluorescent protein. Meanwhile, T cells are discovered to transfer membrane protein to the APCs it recognized. So recognized APCs can be labeled via trogocytosis and later isolated.^24^ Despite advances in these techniques, they are complicated and need to be proved in clinical trials.

#### 2.2.2 Tetramers

Besides using APCs, another commonly used method is to directly present neoantigen by peptide‐MHC (pMHC) tetramers. T cells that bind to pMHC tetramers with fluorescein will be sorted out by FACS.^25^ This method has been applied in diseases such as multiple myeloma and metastatic melanoma.^26^, ^27^ Compared with the aforementioned TMGs and long peptides, a major advantage of pMHC tetramer is that it does not need autologous APCs. Further, this can present every neoantigen equally and successfully. But its liability is it cannot use the natural antigen‐presenting process in the cell and may present antigens that will not appear in the tumor. The way to fabricate pMHC is so sophisticated that it may not be suitable for sorting large amounts of neoantigens. Additionally, it is unable to directly read out the sequence of the neoantigens recognized by T cell.

Facing all these challenges, improvements have been continuously carried out (Figure 2C). Conditional HLA ligands that bind with HLA molecules but can be cleaved upon UV irradiation are developed.^28^, ^29^ The “empty HLA molecules” can then be used to load with antigens of interest. In this way, the time and difficulty of making pMHC are greatly reduced and mass production of pMHC tetramers is feasible. Furthermore, to detect the neoantigen recognized more conveniently, markers on the tetramer are put forward. DNA barcode representing the specific neoantigen is added on the corresponding tetramer, so the neoantigen can be recognized after single‐cell sorting and next‐generation sequencing‐based readout of the barcode.^30^ But this method killed the T cell during DNA sequencing, so it is mostly used in just finding the sequences of neoantigen and the paired TCR. The self‐assembling coding system adds multiple fluorescence in various combinations on same kinds of pMHC tetramer,^31^ T cells are thus marked by unique combinations of color and the antigen can be identified when T cells are alive. But the limited combination of color restricts the number of neoantigens it can identify. Nanoparticle‐barcoded nucleic acid cell sorting (NP‐NACS) method overcomes this problem with a novel DNA barcode,^32^ which has three docking sites for hybridizing dye‐labeled ssDNA in order. T cells combined with tetramers are then sorted in parallel or serial approaches. When hybridized with green, yellow, or red‐labeled ssDNA, the sequence of color is read out and can represent three to the third kinds of neoantigens. By increasing the colors labeled with ssDNA and increasing the docking sites on the DNA barcode, more neoantigen can be selected at the same time.

With the strategies above, tetramers conveniently select neoantigen and T cells responding to it. However, its acceptability is limited by the complexity of construction and defect in antigen processing. Now it is mainly used in confirming the reactivity and specificity of the candidate T cells toward a certain neoantigen, or to pair TCR sequence with cognate antigens.^33^, ^34^

### 2.3 NRT selection

**FIGURE 3:**
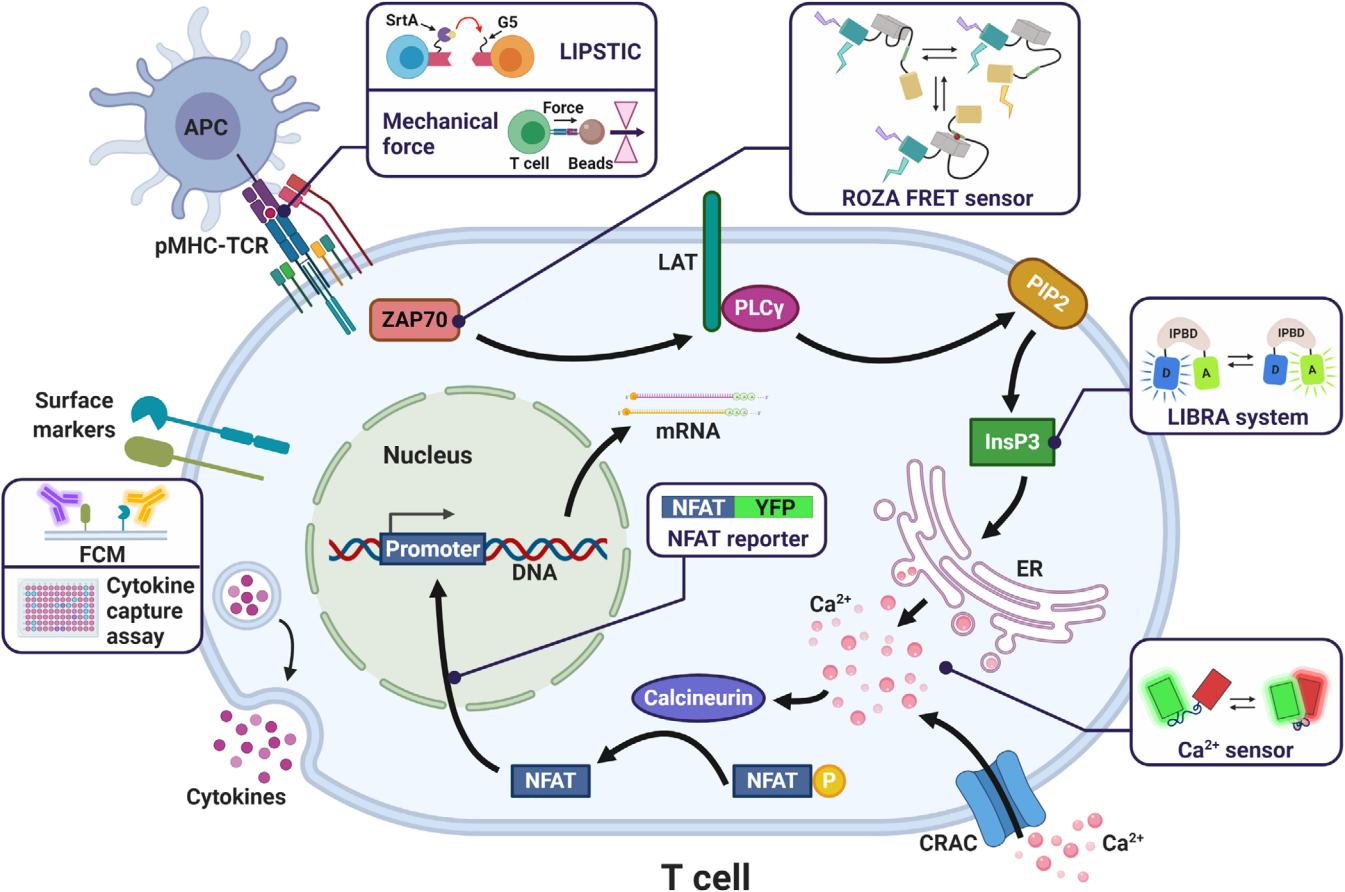
Various approaches applied to select activated T cells. LIPSTIC: an approach that uses a “kiss‐and‐run” interaction between T cell and dendritic cell to transfer substrates and label them; Mechanical force: a system that can detect piconewton‐level intermolecular forces between T cell and pMHC; ROZA FRET sensor: detect activities of ZAP 70 kinase; LIBRA system: contains an IP3 binding domain between CFP and YFP. PLCγ catalyzes PIP2 into IP3. The binding of IP3 influences LIBRA fluorescent signal, which indirectly measures the activity of PLCγ; Ca^2+^ sensor: many types with the same principle that binding of Ca2+ transforms the intramolecular conformation and changes FRET signal; NFAT reporter: NFAT engineered to retain nuclear localization domain but not the DNA‐binding domain is fused with a YFP; Cytokines and cell surface markers can also be used to identify activated T cells Abbreviations: CFP, cyan fluorescent protein; FRET, Förster resonance energy transfer; IP3, inositol trisphosphate; LIBRA, luminous inositol trisphosphate‐binding domain for radiometric analysis; LIPSTIC, labeling immune partnerships by SorTagging intercellular contacts; NFAT, nuclear factor of activated T cells; PIP2, phosphatidylinositol 4,5‐bisphosphate; PLCγ, phosphoinositide phospholipase C‐γ; ROZA, reporter of ZAP‐70 activity; YFP, yellow fluorescent protein; ZAP‐70, zeta‐associated protein of 70 kDa.

Once neoantigens are presented, another challenge is to select the T cells that respond to them (Figure 3).

Based on the TCR‐ligand interaction and subsequent activation of pathways, respectively, there are two main kinds of selecting principles presently. The pMHC tetramer is a typical tool to select T cells by identifying receptor‐ligand interaction as mentioned above. However, it cannot confirm whether the T cells are activated by the antigen or only bind to the antigen. For making up the shortcomings, a robotic microscope system was created to record mechanical force between TCR and antigens.^35^ It detects the acuity of T cell with certain antigen to assess the activated state. Another way is the “Labeling Immune Partnerships by SorTagging Intercellular Contacts” (LIPSTIC) approach.^36^ It intercellularly transfers a specific substrate and labels the T cells and APCs that have interaction. As for the activation of pathways, the classic indicator is cytokines released by the activated T cells, such as interferon (IFN)‐γ.^14^ But it is more suitable to detect the degree of T‐cell cluster response to the neoantigens, because the IFN‐γ is secreted into the microenvironment and it is difficult to trace the single cell.^37^ Cell surface markers are a better choice for identifying stimulated T cells because the cells can be directly selected through flow cytometry. TILs are found to highly express molecules including PD‐1, LAG‐3, TIM‐3, 4‐1BB, CD39, and CD103 on cell surface,^38^, ^39^, ^40^ which are regarded as candidate molecules.^41^ Though the best markers are debatable, the commonly used cell surface marker in NRT is 4‐1BB.^42^ Combinational use of two or three surface markers together may be more accurate to enhance the enrichment of NRTs.^43^ Besides digging deep into surface markers, inspiration is gained from the other perspective of TCR signaling pathway. The associated enzymes are activated, and level of transcriptional factors and important ions will also be upregulated. Specially designed sensors can be used to detect the activity of enzymes such as Zap70 kinase and phospholipase Cγ or the intracellular Ca^2+^ concentration. These designed sensors undergo a conformational switch and change Förster resonance energy transfer (FRET) signal while they are modified by the enzyme or bind to the specific molecules. For example, ROZA FRET sensor consists of a Src homology 2 domain that links to CFP and YFP. While phosphorylated by Zap 70 kinase, the sensor adopts a conformation that conflicts FRET.^44^, ^45^, ^46^, ^47^, ^48^ Truncated nuclear factor of activated T cells‐fluorescent protein (NFAT‐FP) fusion constructs can also be used to reflect the level of activation in the cells. While activated, the reporter will translocate into the nucleus.^49^

Except for selecting T cells from patients’ TILs, healthy people are potential donors. Protocols have been made to identify NRTs from healthy donors’ T cell repertoires.^19^ Regardless of origin, after selection, cell therapy products can be acquired by either expansion and functional modification of natural T cells in vitro, or by expression of the corresponding neoantigen‐reactive TCR in human cell lines.

## 3 OPTIMIZE IN VIVO APPLICATION

**FIGURE 4:**
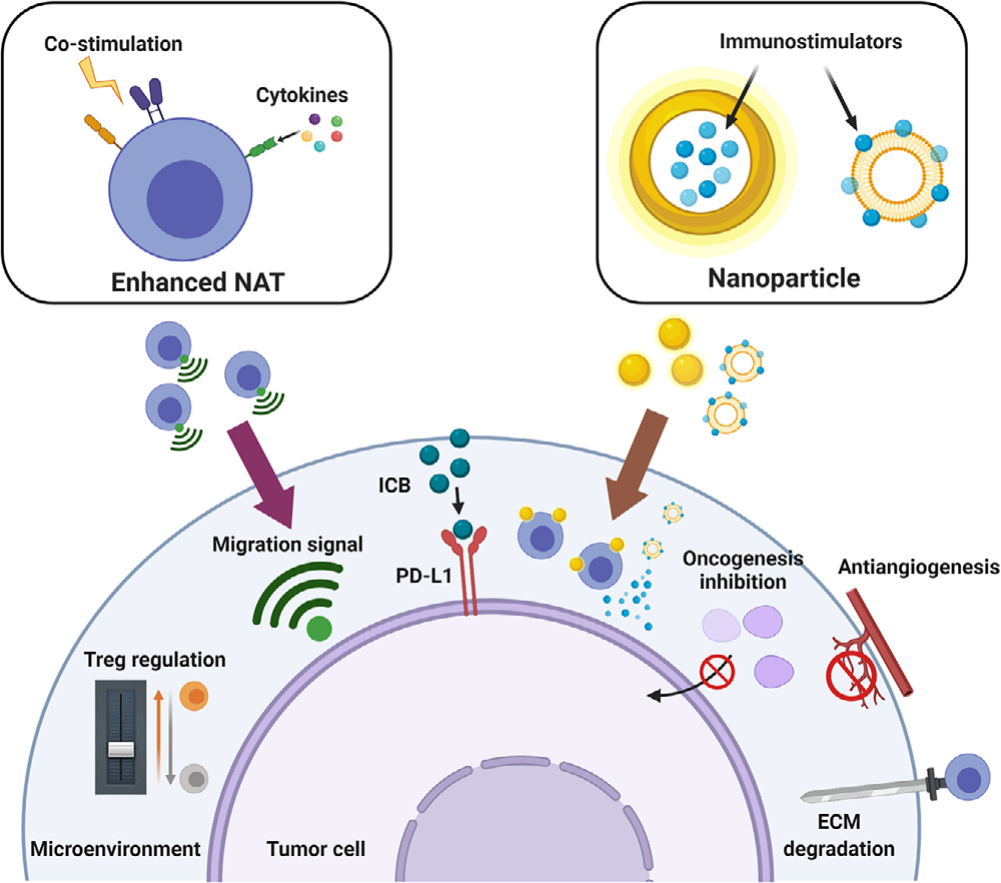
Implementing different potential strategies to strengthen the effect of neoantigen‐reactive T cell immunotherapy. Inadequate T cell function, insufficient number homing to the tumor site, and suppressive microenvironment are the current challenges of therapy. Adequate addition of co‐stimulation molecules and cytokines is able to idealize proliferation and persistence of T cells. With oncogenic pathway inhibition, physical barrier removal, and drug‐receptor migration signals, better local infiltration of T cells can be achieved. Meanwhile, to overcome microenvironment suppression, available methods include immune checkpoint blockade, proper Treg cell regulation, and nanoparticle drug delivery

Preparation of NRTs only lifts half of the veil for NRT therapy. There is still room for improvement of in vivo application (Figure 4).

### 3.1 Augment T cell function

Ideal, persistent T cell activation and proliferation require several signals—TCR signal, co‐stimulation receptor signal, and cytokine receptor signal.^50^ Because T cells with activable TCR have been gathered, the remaining obstacle lies in the latter two.

With regard to co‐stimulation, much progress has been made about 4‐1BB in recent years. 4‐1BB is a costimulatory receptor expressed on activated T cells and other immune cells, responsible for T‐cell memory formation and persistence once bound to 4‐1BB ligand on APCs. Urelumab and utomilumab are two 4‐1BB agonist antibodies under clinical trial but they are not that satisfactory for severe liver toxicity and limited efficacy.^51^ Actually, the immune function and liver toxicity of antibody agonists can be separated by adjusting agonistic activity and the FcγR affinity to an appropriate ratio and a confirmatory antibody agonist was shown to be effective on animal model.^52^ Another method involves a protein that, when bound to the antigen, induces 4‐1BB activation. It avoids liver harm caused by cross‐link of conventional Fc receptor. What's more, combined use of such protein and tumor antigen‐reactive T‐cell bispecific antibodies showed potent antitumor capacity.^53^ There are other classes of co‐stimulation receptors expressed on T‐cell surface, such as CD28, CD27, and ICOS, and their most recent advances are illustrated in another review.^54^ Of course, “Too much water drowned the Miller.” In a CAR‐T study, an excess of CD28 and 4‐1BB co‐stimulation would drive cells to dysfunctional.^55^ Though the conclusion has not been authenticated on human NRTs yet, both exogenous exertion and endogenous design of co‐stimulants should always be administrated within adequate range. Of concern, a latest work on “recyclable CAR” is eye‐catching. During further exploration of its mechanism, researchers found that 4‐1BB domain of remolded CAR contained in endosomes specifically recruits TRAF2 2 (TNF Receptor Associated Factor 2), leading to enhanced downstream signal and better T‐cell persistence.^56^ The finding offers novel idea for NRT optimization.

Similarly, there are seminal discoveries on cytokine assistance. Interleukin‐2 (IL‐2) is a cytokine indispensable for effector T‐cell thriving, but its boosting effect is constrained by concomitant immune suppression and potential toxicity. By remodeling IL‐2 and IL‐2 receptor (IL‐2R), Sockolosky et al introduced synthetic IL‐2‐receptor pairs, enabling mutant IL‐2 and its receptor to specifically bind to each other but not to other natural IL‐2 and IL‐2R.^57^ The new orthogonal complex would pave the way for more accurate and safer NRTs. IL‐23 came into sight in a recent CAR‐T cells study. IL‐23 is composed of two subunits, αp19 and βp40. However, only IL‐23αp19 unit and IL‐23 receptor are upregulated upon T‐cell activation. Transduction of βp40 gene into T cells allows selective proliferation and intensified antitumor power through autocrine IL‐23 signal. It is worthy of mention that the range of action was limited to the activated T cells and IL‐23 preferentially bound to IL‐23 secreting T cells themselves, reducing side effects by sparing bystander cells in tumor.^58^ Another CAR‐T work showed that cytokine combination is feasible. IL‐7 and CCL19 are crucial for T‐cell zone maintenance in lymphoid organs and T cells expressing IL‐7 and CCL19 exhibited great tumor clearance capacity. Moreover, such therapy led dendritic cells and T cells accumulation in tumor sites within mouse models. Multiple benefits also include memory cell formation of both conventional T cells and engineered cells.^59^ Given the role of cytokines in the achievement mentioned above, we suppose single or mixed utilization of cytokines could also be fused into NRT therapy.

### 3.2 Improve homing and infiltration

Though NRT is designed to target neoantigens specifically, inadequacy of T cells homing to the tumor site remains a hindrance to higher T cell therapy response rate. Because sufficient infiltration can enhance other therapies such as ICB,^60^ approaches to drive T cells into tumors are urgently needed.

Oncogenic pathways are available targets because it is found that the overlap of TCR downstream signaling with oncogenic pathway tangles with T‐cell exclusion. BRAF, as a component in MAPK/ERK signal pathway, is commonly found to mutate in quite a few malignancies, and BRAF inhibitors were discovered to be useful in increasing TILs in mouse and human melanoma.^60^, ^61^ Furthermore, releasing growth‐favorable chemokines to impede T cells from cancer site migration is one of their tricks. To help more “assassinators” sneak into the enemy, pro‐tumor chemokine receptors were loaded onto adoptive T cells, and they showed favorable tumor localization and killing capacity.^62^ An endogenous signal‐independent method was developed, which applied a modified G protein‐coupled receptor responding to bioinert drug‐like small molecule, clozapine‐N‐oxide. With a drug‐releasing bead implanted at the tumor site, armed T cells migrated toward the disease focus under guidance.^63^ Extracellular matrix (ECM) and aberrant vasculature are two of physical barriers specific to solid tumors. Previous in vitro manipulation might weaken the penetrability of NRT and other T cell therapies. Engineered T cells expressing enzyme heparinase, which wipes out heparan sulfate proteoglycans, the main components of ECM, have been proved effective.^64^ Also, angiogenesis's counterpart anti‐VEGF can improve T cell homing.^65^ Hence, auxiliary drug administration or remodeling could convoy the future use of NRTs. Surprisingly, physical exercising prior to ACT can increase both quantity and quality of T cells in peripheral blood and after ACT exercise was revealed to benefit T cell infiltration,^66^ which add a fresh dimension to NRT application.

### 3.3 Overcome suppressive microenvironment

Suppressive tumor microenvironment (TME) is a Gordian Knot of T‐cell therapy because various mechanisms are developed to disturb immune efficacy. Here, we review some of the hottest orientations, suggesting therapeutic modalities aimed at overcoming the suppressive state may avail NRT as well.

Cancer cells can directly put brakes on T‐cell activation by expressing immunosuppressive ligands. With the advent of monoclonal antibodies targeting immune checkpoints, ICB is becoming one of the most promising therapeutics. Though it has shown effect on strengthening NRT function in myeloproliferative neoplasms,^67^ more efforts are needed to improve the limited general respond rate. Likewise, PD‐1 plus PD‐L1 blockade is found to promote T‐cell expansion and enhance their function of NRT in pancreatic ductal adenocarcinoma.^68^ In addition to external antibodies, T cells are potential cut‐in points. T cells can be engineered to secrete PD‐1‐blocking single‐chain variable fragments, so that better ICB efficacy and less advert events might be attained.^69^ Of note, PD‐1 on T‐cell surface can be dismantled with CRISPR/Cas9, and PD‐1‐deficient adoptive T cells show superior tumor clearance ability.^70^

Besides the tolerance of tumor cells, other components within TME are involved in immune suppression. It is revealed that TME is infiltrated with substantial regulatory T cells (Treg cell) that curb the immune response and contribute to the immune escape of tumor.^71^ Thus, depletion of Treg cells or downregulation of their inhibiting function can restore robust antitumor immunity. Negative modification of the key transcription factor**—**FOXP3—can result in generation of instable Treg cells with less suppressive function.^72^, ^73^ And blockades of surface molecules (such as CTLA‐4 inhibitor ipilimumab) or immune inhibitory cytokines released by Treg cells enable greater tumor immunity.^74^ Nevertheless, the role of Treg cells is ambiguous in several of malignancies such as colorectal cancer, where higher Treg infiltration turns out to be correlated with better or worse prognosis.^75^ Hence, an equilibrium point of Treg cells should be attained in the combination of NRT applications.

The constitution of TME is quite complex in that it contains fibroblasts, immune cells, neovascular, ECM, and so on, which increase difficulty for precisely assisting antitumor response. Emerging nanoscale drug delivery is worth mention for its specificity, biocompatibility, efficiency boosting, and side effect reducing.^76^ Liposome containing immunostimulatory cytokines is utilized to increase T‐cell function in tumor region. Additionally, cytokine clearance is accelerated compared to free drug given, which avoids systemic toxicities.^77^ Moreover, CAR‐T cells could be equipped with protein nanogels packaging supportive drugs that would be released once T cells recognize their “enemies,” and such T cells would expand in number at the tumor site.^78^ It is interesting that some characteristics of TME can be used against tumors themselves. Molecular and nanoengineering techniques have been applied in cancer immunotherapy. They convert acidic pH, high redox status, hypoxia, and overexpressed enzymes into the stimulus signals so as to release therapeutic medicines or adjuvants at the right place.^79^ Such controllable and specific approach proffers a new view for NRT therapy improvement.

## 4 ELUDE ADVERSE EVENTS

Despite the engineered T cells demonstrate a robust tumor‐killing effect, if not properly controlled, they may elicit dreadful consequences. Currently, many clinical trials of NRT therapy are under way or have been completed, but there is a lack of large‐scale clinical evidence to reveal its safety. CAR‐T, on the other hand, has been tested by a host of clinical trials and clinical applications, and some ways to prevent the adverse events have been proposed.^80^ The experience of CAR‐T may pave the way for a stable and controllable NRT treatment.

Cytokine‐release syndrome (CRS) is one of the most common and severe adverse events of CAR‐T, whereas in the clinical trials of NRT therapy, results were inconsistent in the occurrence of CRS. It is speculated that antigen burden may be positively correlated with the risk of CRS.^81^, ^82^ CRS is an uncontrolled and dysfunctional immune response that results from the continuous release of excessive cytokines (eg, IFN‐γ and TNF‐α) due to aberrant target cell lysis or overactivation of T cells, which further stimulate innate immune cells (eg, macrophages) and endothelial cells.^80^ Depending on the individual variation, potency of infused T cells, and tumor load, clinical manifestations can span from mild symptoms such as arthralgia and fever to hypotension, systemic inflammation, and even shock, multiple organ failure, or death. Clinically available options for the treatment of CRS include supportive therapy, glucocorticoids, as well as targeted therapy with monoclonal antibodies (eg, IL‐6 receptor antagonists Tocilizumab).^83^, ^84^ It is worth mentioning that in the recent COVID‐19, CRS is one of the key pathogenesis in critically ill patients. The artificial‐liver blood‐purification system has been proved to effectively improve the prognosis of COVID‐19 patients by removing inflammatory mediators and blocking cytokine storm.^85^ Although the cytokine profiles of viral infection‐induced and cellular immunotherapy‐induced CRS are not exactly the same,^86^, ^87^ the broad‐spectrum elimination by this approach allows it to be a promising potential for managing severe CRS cases that may occur after NRTs administration.

Another major complication in the clinical use of CAR‐T is neurotoxicity, which occurs independently or in presence of CRS.^88^ However, no significant related symptoms have been found in NRT treatment trials. Some other toxicities such as lymphocytopenia, leukopenia, and anemia may occur, but in general there were no lethal events in previous studies.^81^ As for the other side effects of traditional ACT therapy, such as attacks on normal organs or autoimmune diseases, they are mainly caused by on‐target/off‐tumor toxicities or adoptive cell heterogeneity. The various NRT screening techniques mentioned above have overcome these obstacles. It is important to note that drastic antigen‐targeted attack can also lead to toxicity, such as the tumor lysis syndrome.^89^ Therefore, the issues as the slow‐release delivery system should be considered for the further improvement of NRT therapy while pursuing the vigorous effect.

In addition, more advanced and governable T cells are emerging to solve the potential side effects. As preventive measures, CRS‐promoting proteins such as GMCSF can be genetically edited to block them from the source.^90^ Likewise, CD19‐directed antibody‐γδ‐TCR fusion receptor cells achieve comparable antitumor response but with less cytokine release.^91^ Yet though large amounts of cytokines can trigger a serious of harmful influences, a certain number of cytokines is indispensable for the immune system to abolish the tumor, and this is a balance that needs to be maintained. Of course, not all people have such lethal outcomes as CRS, so interrupt after an unforeseen consequence is also a smart way. Suicide systems are thus developed to ablate the programmed T cells and reduce the biased cytotoxicity. Once a threatening situation happens, the prodrug would be administrated to activate the previously introduced transgene and then results in the termination of adoptive cell function.^92^, ^93^ Another possible strategy is an inducible switch, which controls intracellular signal transduction by sensing exogenous drug signals.^94^ This characteristic endows it with the capacity to manipulate T‐cell activity at will.

## 5 LIMITATIONS OF EXISTING TECHNIQUES

Even compelling as it is, the role of immune system plays in tumor progression and interaction remains to be further explored. Quite a few limitations without ideal solutions hinder further development and application of the current NRT technology.

The first thing to mention is that though it is assumed processed T cells can precisely recognize neoantigens expressed only on the tumor, the cross‐reactivity of TCR‐antigen recognition cannot be ignored. In a cellular immunotherapy study about ovarian cancer, a selected neoantigen‐reactive TCR showed cross‐reactivity against wild‐type counterpart.^95^ And it was observed that MAGE A3‐directed T cells recognized the normal muscle protein Titin in another research,^96^ emphasizing the necessity to reassess the theoretically neoantigen‐reactive TCR. The good thing is that recently launched yeast‐display HLA‐antigen libraries can help identify those “orphan” TCRs,^97^ and at the same time there is a high‐throughput sequencing technique known as TetTCR‐seq that is used to exclude cross‐reactivity and isolate highly conservative receptor sequences.^34^ But further fundamental research to elaborate the underlying mechanisms of immune recognition is required so as to broaden the scope of options for real NRT scanning.

Tumor heterogeneity is one more obstacle on the road to the eradication of tumors. Antigen pool of the tumor is heterogenous but the infused T cells are designed for just a single neoantigen.^98^ In this regard, the selection bias of sequencing samples in preparation phase and partial coverage of TCRs in execution phase will both lead to a decline in the antitumor efficiency. Development of tumor resistance, especially the antigen‐negative escape after a period of treatment, is also very annoying. The involved mechanisms are manifested as downregulation or loss of target antigen, or errors in antigen presentation process such as defect in proteasomes, transporters, or MHCs.^99^ It is particularly challenging for the monoclonal T cell subsets because tumor mutations are more prone to occur in such selection pressure. Indeed, hybrid T cells targeted to various neoantigen‐MHC combinations had been proved to be more aggressive against melanoma and prostate cancer in vitro.^100^ Such progressive findings add weight to the idea of cocktail therapy, that is, compound NAT cell formulations plus other complementary therapeutic approaches (eg, surgery, chemotherapy, radiotherapy, other immunotherapy, etc), which possesses a stronger ability specific to “panoramic” tumor and impervious to antigen evasion. More clinical explorations are needed to extend broader antitumor activity and boost the benefits afforded by NAT therapy.

Irrefutably, given the state of art, other issues such as the time and cost of the techniques itself are a definite barrier. We will cover it in the following section and not repeat it here.

## 6 DISCUSSION

NRT therapy is on the rise due to its good specificity, significant antitumor activity, and relatively mild side effects. Because it is still in rookie stage, there is not much headway on it yet. Through the above review, we present the overview of the NRT therapy and summarize the current progresses made on its forward march. Moreover, by harnessing the existing research in basic immunology and other immunotherapy‐related field, we demonstrate several other possible obstacles in front of NRT application and propose the countermeasures correspondingly. As a matter of fact, however, we have only scratched the surface of what NRT therapy is all about, and there are deeper realms for us to excavate.

Nowadays the identification of neoantigens mainly relies on methods at the genome scale, such as whole exome sequencing. Blossom of multiomics and the deep learning will enable us to unearth more potential neoantigens. The concept of tumor antigen may also be extended into misfolded proteins, nucleic acids, exosomes, and other forms. A parallel step is to develop noninvasive liquid biopsy tools for early screening and intervention, avoiding the trouble of surgical sampling. As to the screening and modification of T cells, tremendous engineering approaches are created, but there is no consensus on which is the optimal solution yet. Two directions, one is to induce the differentiation of immune stem cells into target cells and the other is to rejuvenate the available cells to achieve long‐lasting effects, are both bonanzas full of possibility. For a proper cellular formulation, a comprehensive understanding is needed to clarify the role of diverse cells in the antitumor process. Further clinical trials can provide supportive evidence for the therapeutic window of different modalities. The exploitable space in TME is boundless, and omnifarious research directions and novel concepts are emerging. Recent reports on the pyroptosis‐induced inflammation in antitumor immunity may offer such a new pathway.^101^, ^102^ Interdisciplinary approaches, such as photoinduced or magnetically targeted delivery system, would also exert power to overcome some biological constraints. Relevant progress has been finely summarized elsewhere,^79^ and it is guiding immunotherapy to a more intelligent and microscopic direction. Another desired form of cellular therapy is to screen, modify, and amplify in vivo through gene editing and nanoscale manipulation platforms, which is likely to be the following milestone. Although the technical cost and time required to implement personalized procedure is a controversial point, some tend to obtain “off the shelf” target to unify the production lines and quickly produce cell products with low expenditure. However, based on previous experience in tumor treatment, the “made to measure” therapy may be the better option for patients. Gradual replacement of manual labor by robots and maturity of streamlined procedure will shorten time and reduce the costs to acceptable levels. Overall, though there are still many hurdles on the way, once crossed, neoantigen‐reactive cell therapy will sparkle as a mighty weapon for those health threats.

## CONFLICT OF INTEREST

The authors declare that this work was conducted in the absence of any commercial or financial relationships that could be construed as a potential conflict of interest. Bin Li is a co‐founder of Biotheus Inc and the chairman of its scientific advisory board.

## AUTHOR CONTRIBUTIONS

YZ contributed to the central idea and coordinated the writing of the manuscript. YZ, YQ, and ZL wrote the manuscript and made the figures and the table. YZ, YQ, ZL, YL, and BL read, discussed, and revised the manuscript. All authors listed have made a substantial, direct, and intellectual contribution to the work, and approved it for publication.

## ETHICS STATEMENT

Not applicable.
